# Self-Reported Symptoms of SARS-CoV-2 Infection in a Nonhospitalized Population in Italy: Cross-Sectional Study of the EPICOVID19 Web-Based Survey

**DOI:** 10.2196/21866

**Published:** 2020-09-18

**Authors:** Fulvio Adorni, Federica Prinelli, Fabrizio Bianchi, Andrea Giacomelli, Gabriele Pagani, Dario Bernacchia, Stefano Rusconi, Stefania Maggi, Caterina Trevisan, Marianna Noale, Sabrina Molinaro, Luca Bastiani, Loredana Fortunato, Nithiya Jesuthasan, Aleksandra Sojic, Carla Pettenati, Marcello Tavio, Massimo Andreoni, Claudio Mastroianni, Raffaele Antonelli Incalzi, Massimo Galli

**Affiliations:** 1 Epidemiology Unit Institute of Biomedical Technologies National Research Council Segrate (MI) Italy; 2 Department of Environmental Epidemiology and Disease Registries Institute of Clinical Physiology National Research Council Pisa Italy; 3 Infectious Diseases Unit Department of Biomedical and Clinical Sciences L. Sacco University of Milan Milan Italy; 4 Aging Branch Neuroscience Institute National Research Council Padoa Italy; 5 Geriatric Unit Department of Medicine (DIMED) University of Padova Padoa Italy; 6 Epidemiology and Health Research Laboratory Institute of Clinical Physiology National Research Council Pisa Italy; 7 Division of Infectious Diseases Azienda Ospedaliero Universitaria Ospedali Riuniti Ancona Italy; 8 Infectious Diseases Clinic Department of System Medicine Tor Vergata University of Rome Rome Italy; 9 Public Health and Infectious Disease Department Sapienza University Rome Italy; 10 Unit of Geriatrics Department of Medicine Biomedical Campus of Rome Rome Italy

**Keywords:** SARS-CoV-2, COVID-19, voluntary respondents, web-based survey, self-reported symptom, nasopharyngeal swab testing, cross-sectional

## Abstract

**Background:**

Understanding the occurrence of symptoms resembling those of severe acute respiratory syndrome coronavirus 2 (SARS-CoV-2) in a large nonhospitalized population at the peak of the epidemic in Italy is of paramount importance; however, data are currently scarce.

**Objective:**

The aims of this study were to evaluate the association of self-reported symptoms with SARS-CoV-2 nasopharyngeal swab (NPS) test results in nonhospitalized individuals and to estimate the occurrence of symptoms associated with coronavirus disease (COVID-19) in a larger nontested population.

**Methods:**

EPICOVID19 is a self-administered cross-sectional voluntary web-based survey of adults throughout Italy who completed an anonymous questionnaire in the period of April 13 to 21, 2020. The associations between symptoms potentially related to SARS-CoV-2 infection and NPS results were calculated as adjusted odds ratios (aORs) with 95% CIs by multiple logistic regression analysis controlling for age, sex, education, smoking habits, and number of comorbidities. Thereafter, for each symptom and for combinations of the symptoms, we calculated the sensitivity, specificity, accuracy, and areas under the curve (AUCs) in a receiver operating characteristic (ROC) analysis to estimate the occurrence of COVID-19–like infection in the nontested population.

**Results:**

A total of 171,310 people responded to the survey, of whom 102,543 (59.9%) were women; mean age 47.4 years. Out of the 4785 respondents with known NPS test results, 4392 were not hospitalized. Among the 4392 nonhospitalized respondents, those with positive NPS tests (856, 19.5%) most frequently reported myalgia (527, 61.6%), olfactory and taste disorders (507, 59.2%), cough (466, 54.4%), and fever (444, 51.9%), whereas 7.7% were asymptomatic. Multiple regression analysis showed that olfactory and taste disorders (aOR 10.3, 95% CI 8.4-12.7), fever (aOR 2.5, 95% CI 2.0-3.1), myalgia (aOR 1.5, 95% CI 1.2-1.8), and cough (aOR 1.3, 95% CI 1.0-1.6) were associated with NPS positivity. Having two to four of these symptoms increased the aOR from 7.4 (95% CI 5.6-9.7) to 35.5 (95% CI 24.6-52.2). The combination of the four symptoms showed an AUC of 0.810 (95% CI 0.795-0.825) in classifying positive NPS test results and then was applied to the nonhospitalized and nontested sample (n=165,782). We found that 7739 to 20,103 of these 165,782 respondents (4.4% to 12.1%) had experienced symptoms suggestive of COVID-19 infection.

**Conclusions:**

Our results suggest that self-reported symptoms are reliable indicators of SARS-CoV-2 infection in a pandemic context. A nonnegligible number of symptomatic respondents (up to 12.1%) were undiagnosed and potentially contributed to the spread of the infection.

**Trial Registration:**

ClinicalTrials.gov NCT04471701; https://clinicaltrials.gov/ct2/show/NCT04471701

## Introduction

The outbreak of severe acute respiratory syndrome coronavirus 2 (SARS-CoV-2), which started in late December 2019 in Hubei Province in China, caused millions of cases of coronavirus disease (COVID-19) worldwide in just a few months and evolved into a pandemic [1,2]. As of June 25, 2020, there were 239,706 confirmed cases of COVID-19 in Italy and 34,678 reported deaths [3].

It is worth noting that only approximately 20% of patients infected with SARS-CoV-2 require hospital care [4]. The vast majority of patients experience mild or subclinical forms of the disease that do not require hospital admission [5], and a relatively high percentage of patients (40% to 45%) remain asymptomatic [6].

Patients with SARS-CoV-2 frequently report fever, upper respiratory symptoms, myalgia, headache, and gastrointestinal disturbances [4,7] as well as olfactory and taste disorders [8]. However, the prevalence of COVID-19–related symptoms in the population of nonhospitalized patients has not been well investigated [9,10]. Early recognition of the conditions attributable to the infection is of paramount importance. This is particularly relevant for promptly identifying not only cases with severe clinical courses but also cases with milder symptomatology who can spread the infection and who must be immediately quarantined while testing and contact tracing is conducted.

This study is based on EPICOVID19, an anonymized self-administered web-based survey aimed at estimating the number of suspected cases of COVID-19 and investigating the role of potential determinants of SARS-CoV-2 infection in a large sample of respondents living in Italy during the lockdown, which started in Italy on March 9, 2020. The aims of this paper are to evaluate the association of self-reported symptoms with SARS-CoV-2 nasopharyngeal swab (NPS) test results in nonhospitalized individuals and to estimate the occurrence of COVID-19–like symptoms in the nontested population.

## Methods

### Study Design and Setting

EPICOVID19 is a national Italian internet-based survey that was conducted using a cross-sectional research design by a working group dedicated to collaborative public health research related to SARS-CoV-2. The survey was launched on April 13, 2020, and it targeted adult volunteers living in Italy during the lockdown. The study was registered (ClinicalTrials.gov NCT04471701).

### Recruitment

To enroll as many participants as possible, the survey was promoted using social media (Facebook, Twitter, Instagram, and WhatsApp), press releases, web pages, local radio and television stations, and institutional websites that called upon volunteers to contact the study website [11]. The inclusion criteria were age >18 years; access to a mobile phone, computer, or tablet with internet connectivity; and provision of web-based consent to participate in the study.

### Development of the Web-Based Questionnaire

EPICOVID19 was developed by the working group after a literature review of existing research into COVID-19, starting with the World Health Organization protocols [12], and of the standard and validated instruments previously used to investigate severe acute respiratory syndrome (SARS) and Middle Eastern respiratory syndrome (MERS) [13,14].

The questionnaire was adapted to the national context and implemented using the European Commission’s open-source official EUSurvey management tool [15]. The participants were asked to complete the self-administered 38-item questionnaire, which mainly contained mandatory and closed questions divided into 6 sections: 1) sociodemographic data; 2) clinical evaluation; 3) personal characteristics and health status; 4) housing conditions; 5) lifestyle; and 6) behaviors following the lockdown (see Multimedia Appendix 1).

### Data Collection and Variables

For the purposes of this study, we analyzed a subset of data collected between April 13 and 21, 2020. The sociodemographic information included sex (male and female), age (18 to 30, 30 to 39, 40 to 49, 50 to 59, 60 to 69, 70 to 79, and ≥80 years), educational level (primary school or less, middle or high school, and university degree or postgraduate degree), and occupational status (unemployed, employed, retired, student, and other). Smoking habits were classified as never smoked, former smoker, and current smoker. A new variable was created by summing the chronic conditions reported by participants, including lung diseases, heart diseases, hypertension, kidney diseases, immune system diseases, tumors, metabolic diseases, liver diseases, and depression and anxiety (categorized as no, 1, 2, or >3 comorbidities). The SARS-CoV-2–related symptoms included fever >37.5 degrees Celsius for at least three consecutive days; headache, chest pain, myalgia, olfactory and taste disorders, shortness of breath, and heart palpitations; gastrointestinal disturbances, including nausea, vomiting and diarrhea; conjunctivitis; and sore throat, rhinorrhea, and cough (all dichotomized as present/absent). The month of onset of the first symptoms (February/March/April 2020), NPS test results (categorized as not performed, performed with a negative result, performed with a positive result, and performed with an unknown result), and hospitalization for confirmed or suspected SARS-CoV-2 infection (dichotomized as yes/no) were also collected.

### Study Group Definitions

To achieve the aims of this study, we defined three study samples:

1. Sample A, including the total population of respondents (N=171,310).
2. Subsample B, including nonhospitalized individuals and individuals who reported NPS tests with known results (n=4392).
3. Subsample C, including the nonhospitalized and nontested individuals (n=165,782).

### Statistical Analysis

The continuous variables were expressed as mean (SD), and the categorical variables were expressed as counts and percentages. The chi-square test and one-way analysis of variance were used to compare the characteristics of respondents by NPS test results (sample A). The geographical coverage of the sample was evaluated by calculating the response rates by Italian region standardized by the number of residents aged >18 years on January 1, 2019 [16]. When analyzing subsample B, we calculated the matrix of pairwise tetrachoric correlations of self-reported symptoms, given the dichotomous nature of these variables. Crude and adjusted logistic regression models, controlling for age, sex, education, smoking habit, and number of comorbidities, were applied to assess the association between self-reported symptoms and SARS-CoV-2 positive NPS test versus negative NPS test by estimating the adjusted odds ratios (aORs) and 95% CIs. Subsequently, a numerical variable including all the symptoms significantly associated with NPS positivity was created and included in the logistic regression model instead of the single symptoms. Age- and sex-stratified analyses were also performed. In a sensitivity analysis, we excluded the respondents who reported February as the month of symptom onset to avoid possible confounding by influenza-like illness (the peak of the Italian influenza season in 2019-2020 occurred from January 27 to February 2) [17]. Finally, after assessing the sensitivity, specificity, and area under the curve (AUC) in a receiver operating characteristic (ROC) analysis, the symptoms significantly associated with positive NPS test results were combined as a proxy of COVID-19–like infection in subsample C. All the statistical analyses were carried out using SPSS version 25 (IBM Corp) and STATA version 15.0 (StataCorp LP). Two-tailed *P* values <.05 were considered statistically significant.

### Ethics and Consent Form

The Ethics Committee of the Istituto Nazionale per le Malattie Infettive I.R.C.C.S. Lazzaro Spallanzani (Protocol No. 70, 12/4/2020) approved the EPICOVID19 study protocol. When participants first accessed the web-based platform, they were informed of the purpose of the study, the data to be collected, and the methods of storage, and they filled in the informed consent form. The planning, conduction, and reporting of the studies was in line with the Declaration of Helsinki as revised in 2013. Data were handled and stored in accordance with the European Union General Data Protection Regulation (EU GDPR) 2016/679, and data transfer was safeguarded by encrypting/decrypting and password protection.

## Results

### Characteristics of the Respondents

Table 1 summarizes the characteristics of the 171,310 respondents who completed the survey between April 13 and 21, 2020 (sample A). The respondents were prevalently female (102,543/171,310, 59.9%); the mean age of the female respondents was 46.8 years (SD 14.2) and that of the male respondents was 48.2 years (SD 15.0). Of the 171,310 respondents, 104,583 (61.0%) had a university degree or post-graduate qualification, and most were regularly employed (119,585, 69.8%). Smokers and ex-smokers accounted for 72,929/171,310 (42.6%) of the respondents, including 40,949/102,543 (39.9%) of the female respondents and 31,980/68,767 (46.5%) of the male respondents. About two-thirds of the 171,310 respondents (111,181, 64.9%) had no chronic conditions, and the vast majority (165,993, 96.9%) did not undergo NPS testing for SARS-CoV-2. Of the 5317/171,310 respondents (3.1%) who did undergo NPS testing, 1135 (21.3%) tested positive, 3650 (68.6%) tested negative, and 532 (10.0%) had not received the results at the time of completing the questionnaire. A total of 170,700 respondents were nonhospitalized.

**Table 1:** Characteristics of the survey respondents by sex (sample A).

Characteristic		Sex at birth				Total (N=171,310)
		Female (n=102,543, 59.9%)		Male (n=68,767, 40.1%)		
Age (years), mean (SD)		46.8 (14.2)		48.2 (15.0)		47.4 (14.5)
**Age (years), n (%)**						
	18-30	13,538 (13.2)		8611 (12.5)		22,149 (12.9)
	30-39	21,002 (20.5)		13,351 (19.4)		34,353 (20.1)
	40-49	22,907 (22.3)		14,412 (21.0)		37,319 (21.8)
	50-59	23,815 (23.2)		14,941 (21.7)		38,756 (22.6)
	60-69	16,088 (15.7)		11,710 (17.0)		27,798 (16.2)
	70-79	4386 (4.3)		4938 (7.2)		9324 (5.4)
	≥80	807 (0.8)		804 (1.2)		1611 (0.9)
**Education, n (%)**						
	Primary school or less	5036 (4.9)		4005 (5.8)		9041 (5.3)
	Middle or high school	33,049 (32.2)		24,637 (35.8)		57,686 (33.7)
	University degree or post-graduate degree	64,458 (62.9)		40,125 (58.3)		104,583 (61.0)
**Occupational status, n (%)**						
	Unemployed	5632 (5.5)		2136 (3.1)		7768 (4.5)
	Employed	70,577 (68.8)		49,008 (71.3)		119,585 (69.8)
	Retired	12,281 (12.0)		10,594 (15.4)		22,875 (13.4)
	Student	7196 (7.0)		4757 (6.9)		11,953 (7.0)
	Other	6857 (6.7)		2272 (3.3)		9129 (5.3)
**Smoking habit, n (%)**						
	Never smoked	61,594 (60.1)		36,787 (53.5)		98,381 (57.4)
	Former smoker	22,017 (21.5)		18,986 (27.6)		41,003 (23.9)
	Current smoker	18,932 (18.5)		12,994 (18.9)		31,926 (18.6)
**Number of comorbidities, n (%)**						
	None	66,294 (64.6)		44,887 (65.3)		111,181 (64.9)
	One	27,016 (26.3)		17,562 (25.5)		44,578 (26.0)
	Two	7099 (6.9)		4841 (7.0)		11,940 (7.0)
	Three or more	2134 (2.1)		1477 (2.1)		3611 (2.1)
**Molecular test for SARS-CoV-2^a^, n (%)**						
	Not performed	99,084 (96.6)		66,909 (97.3)		165,993 (96.9)
	Performed, with a negative result	2440 (2.4)		1210 (1.8)		3650 (2.1)
	Performed, with a positive result	668 (0.7)		467 (0.7)		1135 (0.7)
	Performed, with an unknown result	351 (0.3)		181 (0.3)		532 (0.3)
Hospitalized for suspected/confirmed SARS-CoV-2 infection, n (%)		328 (0.3)		282 (0.4)		610 (0.4)
Not hospitalized with known molecular test results, n (%)		2931 (2.9)		1461 (2.1)		4392 (2.6)

^a^SARS-CoV-2: severe acute respiratory syndrome coronavirus 2.

Of the 171,310 respondents, 610 (0.35%) said that they had been hospitalized between February 1 and April 21, 2020, including 399 of the 5317 respondents (7.5%) who were tested for SARS-CoV-2 infection (Supplementary Table S1 in Multimedia Appendix 2). Female and younger respondents were less likely to report positive NPS tests, whereas respondents who had a lower level of education or were retired more frequently reported positive NPS tests. Current smokers were less prevalent among the respondents with positive NPS tests (108/1135, 9.5%).

### Geographical Coverage

**Figure 1:**
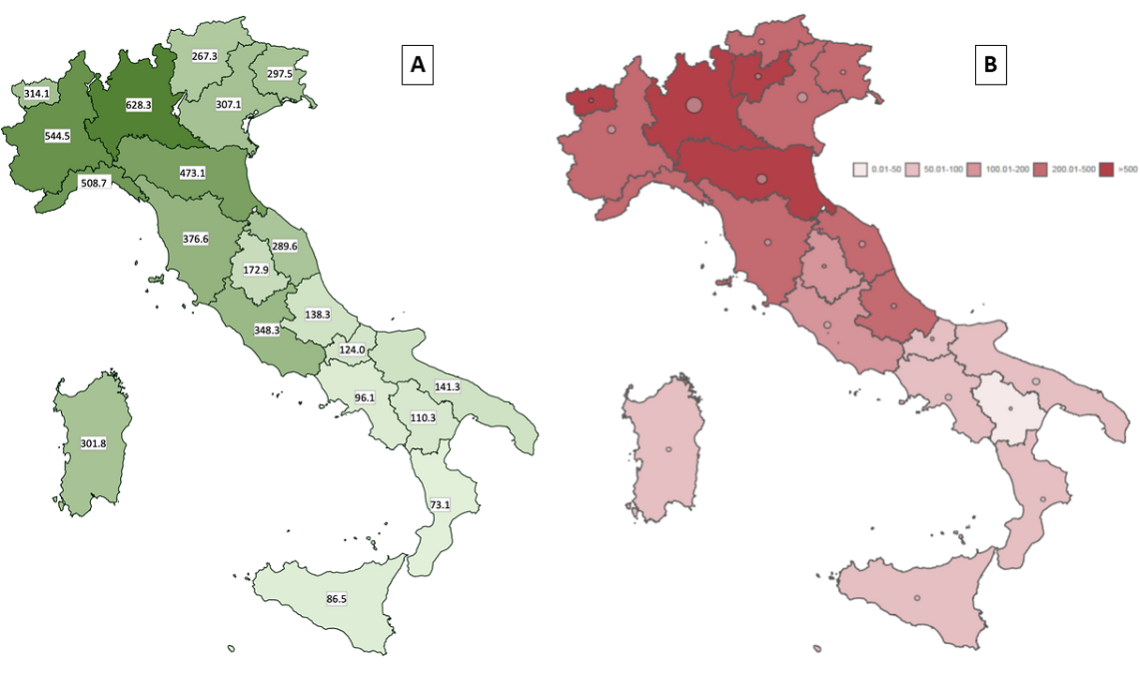
Comparison of the survey response rates and the incidence of severe acute respiratory syndrome coronavirus 2 (SARS-CoV-2) infection per 100,000 inhabitants by Italian region. A: Response rates × 100,000. B: Incidence rates of SARS-CoV-2 × 100,000.

Although the survey lacked a formal sampling strategy, a large number of participants were reached throughout Italy. Figure 1 shows the standardized response rates and the incidence of SARS-CoV-2 infection per 100,000 inhabitants by Italian region as of April 23, 2020 [16,18]. As expected, response rates were higher in the northern regions (Lombardy and Piedmont) and reflected the incidence of confirmed cases at that time.

### Self-Reported Symptoms

**Figure 2:**
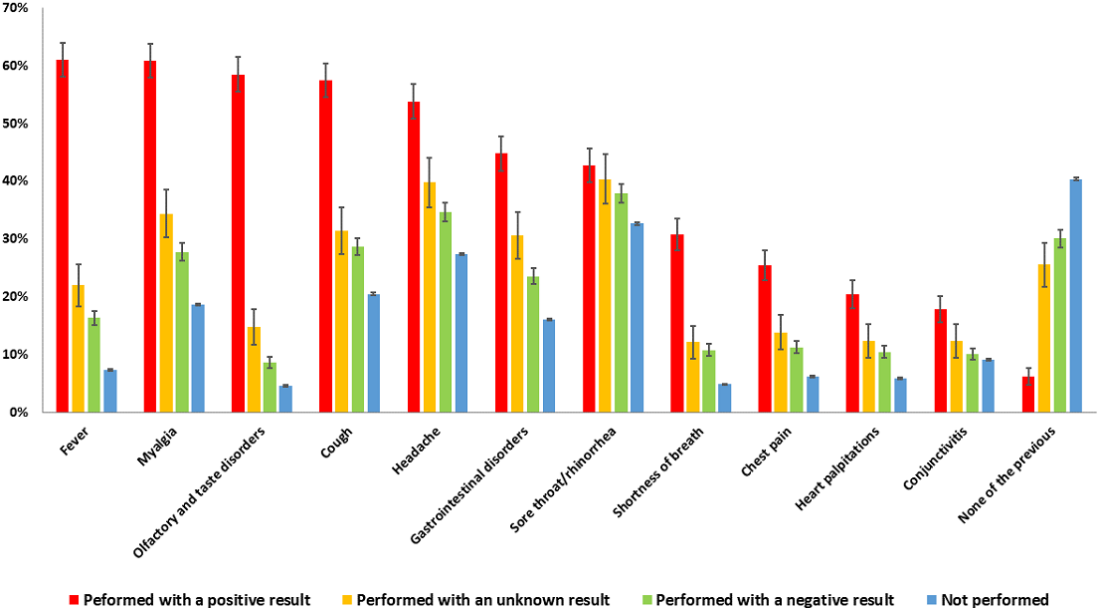
Self-reported symptoms by 171,310 survey respondents. Error bars are ±2*standard error (normal approximation).

Figure 2 shows that 68,337 of the 171,310 respondents (39.9%) indicated no symptoms. The most frequently reported symptoms were sore throat/rhinorrhea (56,324/171,310, 32.9%), headache (47,521/171,310, 27.7%), myalgia (32,856/171,310, 19.2%), gastrointestinal disturbances (28,212/171,310, 16.5%), conjunctivitis (15,872/171,310, 9.3%), and fever (13,752/171,310, 8.0%) (sample A). The absence of symptoms was less frequent among respondents with positive NPS tests than among those with negative tests (70/1135, 6.2%, vs 1100/3650, 30.1%), and there were also notable between-group differences in the frequency of fever (692/1135, 61.0%, vs 600/3650, 16.4%), olfactory and taste disorders (664/1135, 58.5%, vs 319/3650, 8.7%), myalgia (690/1135, 60.8%, vs 1015/3650, 27.8%), cough (653/1135, 57.5%, vs 1048/3650, 28.7%), headache (611/1135, 53.8%, vs 1265/3650, 34.7%), and gastrointestinal symptoms (508/1135, 44.8%, vs 863/3650, 23.6%). Table 2 shows that among 102,973 symptomatic respondents, the mean number of symptoms was 5.05 among respondents with positive NPS tests, 3.55 among respondents with unknown results, 3.16 among respondents with negative results, and 2.57 among respondents who did not undergo the molecular test (*P*<.001).

**Table 2:** Symptoms reported by survey respondents (n=102,973) and mean numbers of symptoms per symptomatic respondent based on molecular testing status.

Number of self-reported symptoms	Test performed with a positive result	Test performed with an unknown result	Test performed with a negative result	Test not performed	Total
Total (n)	5379	1407	8053	254,714	269,553
Per symptomatic respondent (mean)	5.05	3.55	3.16	2.57	2.62

In the tetrachoric correlation analysis between symptoms (Supplementary Table S2, Multimedia Appendix 2) performed in subsample B, values of the correlation coefficient >.6 were observed in the subgroup of symptoms including fever, olfactory and taste disorders, cough, and myalgia, while values <.3 were mainly observed for sore throat/rhinorrhea and conjunctivitis. In the same subsample B, from univariate and multiple logistic regression analysis controlling for sex, age, education, smoking habit, and number of comorbidities, all the considered symptoms were found to be positively associated with a positive NPS test (Table 3). In the final multiple regression model, with all symptoms included, olfactory and taste disorders (aOR 10.32, 95% CI 8.39-12.70), fever (aOR 2.46, 95% CI 1.98-3.05), myalgia (aOR 1.45, 95% CI 1.17-1.80), and cough (aOR 1.28, 95% CI 1.03-1.58) were found to be significantly associated with a positive NPS test. The odds of a positive test also increased with each additional year of age (aOR 1.02, 95% CI 1.01-1.03) and with male sex (aOR 1.34, 95% CI 1.11-1.63), whereas current smoking (aOR 0.66, 95% CI 0.50-0.87) was associated with decreased odds (data not shown). After adding the composite variables of fever, myalgia, cough, and olfactory and taste disorders to the model and simultaneously adjusting for the other symptoms, we found a strong positive and statistically significant association. The corresponding aORs for the presence of one, two, three, and four of these symptoms were 2.66 (95% CI 2.03-3.49), 7.35 (95% CI 5.57-9.70), 18.55 (95% CI 13.77-24.97), and 35.50 (95% CI 24.60-51.24), respectively.

**Table 3:** Odds ratios of positive molecular tests in nonhospitalized respondents with known molecular test results (n=4392, subsample B).

Symptom		Negative (n=3536, 80.5%), n (%)	Positive (n=856, 19.5%), n (%)	Model 1, OR^a^ (95% CI)^b^	Model 2, aOR^c^ (95% CI)^d^	Model 3, aOR (95% CI)^e^	*P* value^f^
Fever		518 (14.6)	444 (51.9)	6.28 (5.33-7.39)	6.08 (5.15-7.17)	2.46 (1.98-3.05)	<.001
Myalgia		961 (27.2)	527 (61.6)	4.29 (3.67-5.02)	4.33 (3.69-5.07)	1.45 (1.17-1.80)	.001
Olfactory and taste disorders		291 (8.2)	507 (59.2)	16.20 (13.51-19.42)	16.98 (14.07-20.48)	10.32 (8.39-12.70)	<.001
Cough		984 (27.8)	466 (54.4)	3.10 (2.66-3.61)	3.09 (2.65-3.61)	1.28 (1.03-1.58)	.02
Shortness of breath		335 (9.5)	182 (21.3)	2.58 (2.12-3.15)	2.63 (2.15-3.23)	0.89 (0.67-1.18)	.40
Chest pain		386 (10.9)	206 (24.1)	2.59 (2.14-3.12)	2.61 (2.15-3.16)	0.92 (0.70-1.20)	.54
Heart palpitations		354 (10.0)	165 (19.3)	2.15 (1.75-2.63)	2.21 (1.80-2.72)	0.93 (0.70-1.23)	.61
Gastrointestinal disturbances		817 (23.1)	382 (44.6)	2.68 (2.30-3.13)	2.82 (2.40-3.30)	1.20 (0.98-1.48)	.08
Conjunctivitis		351 (9.9)	156 (18.2)	2.02 (1.65-2.48)	2.07 (1.68-2.55)	1.11 (0.85-1.45)	.45
Sore throat/rhinorrhea		1332 (37.7)	415 (48.5)	1.56 (1.34-1.81)	1.64 (1.40-1.91)	0.87 (0.71-1.07)	.18
Headache		1213 (34.3)	485 (56.7)	2.50 (2.15-2.91)	2.64 (2.26-3.09)	1.18 (0.95-1.45)	.13
**Number of symptoms^g^**							
	None	1931 (54.6)	118 (13.8)	1	1	1	N/A^h^
	One	854 (24.1)	133 (15.5)	2.55 (1.96-3.31)	2.61 (2.01-3.39)	2.66 (2.03-3.49)	<.001
	Two	441 (12.5)	185 (21.6)	6.86 (5.33-8.84)	7.06 (5.47-9.12)	7.35 (5.57-9.70)	<.001
	Three	222 (6.3)	239 (27.9)	17.62 (13.58-22.86)	17.86 (13.71-23.27)	18.55 (13.77-24.97)	<.001
	All	88 (2.5)	181 (21.1)	33.66 (24.56-46.14)	34.02 (24.71-46.85)	35.50 (24.60-51.24)	<.001

^a^OR: odds ratio.

^b^Crude ORs.

^c^aOR: adjusted odds ratio.

^d^Controlling for sex, age, education, smoking habit, and number of comorbidities.

^e^Controlling for sex, age, education, smoking habit, and number of comorbidities, including all symptoms.

^f^*P* values refer to model 3.

^g^Ordinal variable summing the presence of fever, myalgia, cough, and olfactory and taste disorders.

^h^N/A: not applicable.

Excluding the respondents who indicated that their first symptom appeared in February from the sensitivity analysis did not substantially change the results (Supplementary Table S3 in Multimedia Appendix 2).

Tables 4 and 5 show the results of the sex- and age-stratified multiple regression analyses. Olfactory and taste disorders were more closely associated with the odds of a positive test in female respondents (aOR 12.10, 95% CI 9.35-15.67) and respondents aged <50 years (aOR 15.88, 95% CI 12.10-20.84), whereas fever was more closely associated with a positive NPS test in male respondents (aOR 3.90, 95% CI 2.72-5.59) and respondents aged >50 years (aOR 3.46, 95% CI, 2.50-4.78).

**Table 4:** Sex-specific adjusted odds ratios of positive molecular tests in nonhospitalized survey respondents with known molecular test results (n=4392, subsample B).

Symptom		Female respondents (n=2931, 66.7%)				Male respondents (n=1461, 33.3%)					
		Negative test (n=2376, 81.1%), n (%)	Positive test (n=555, 18.9%), n (%)	aOR^a,b^ (95% CI)	*P* value	Negative test (n=1160, 79.4%), n (%)		Positive test (n=301, 20.6%), n (%)		aOR (95% CI)	*P* value
Fever		341 (14.4)	272 (49.0)	1.87 (1.42-2.46)	<.001	177 (15.3)		172 (57.1)		3.90 (2.72-5.59)	<.001
Myalgia		674 (28.4)	352 (63.4)	1.42 (1.08-1.87)	.01	287 (24.7)		175 (58.1)		1.42 (0.99-2.06)	.06
Olfactory and taste disorders		210 (8.8)	357 (64.3)	12.10 (9.35-15.67)	<.001	81 (7.0)		150 (49.8)		8.58 (5.92-12.43)	<.001
Cough		667 (28.1)	306 (55.1)	1.34 (1.03-1.74)	.03	317 (27.3)		160 (53.2)		1.13 (0.79-1.62)	.50
Shortness of breath		239 (10.1)	131 (23.6)	0.88 (0.63-1.23)	.45	96 (8.3)		51 (16.9)		0.91 (0.54-1.55)	.74
Chest pain		276 (11.6)	154 (27.7)	1.04 (0.76-1.44)	.79	110 (9.5)		52 (17.3)		0.72 (0.43-1.18)	.19
Heart palpitations		279 (11.7)	129 (23.2)	0.91 (0.66-1.27)	.60	75 (6.5)		36 (12.0)		1.10 (0.62-1.94)	.75
Gastrointestinal disturbances		587 (24.7)	266 (47.9)	1.14 (0.88-1.48)	.34	230 (19.8)		116 (38.5)		1.34 (0.94-1.92)	.11
Conjunctivitis		244 (10.3)	112 (20.2)	1.19 (0.86-1.64)	.29	107 (9.2)		44 (14.6)		1.00 (0.61-1.63)	.99
Sore throat/rhinorrhea		950 (40.0)	279 (50.3)	0.77 (0.60-0.99)	.04	382 (32.9)		136 (45.2)		1.06 (0.75-1.49)	.76
Headache		900 (37.9)	338 (60.9)	1.09 (0.84-1.42)	.50	313 (27.0)		147 (48.8)		1.33 (0.93-1.90)	.11
**Number of symptoms^c^**											
	None	1288 (54.2)	73 (13.2)	1	N/A^d^	643 (55.4)		45 (15.0)		1	N/A^d^
	One	566 (23.8)	85 (15.3)	2.81 (2.00-3.96)	<.001	288 (24.8)		48 (15.9)		2.39 (1.53-3.72)	<.001
	Two	306 (12.9)	112 (20.2)	7.13 (5.00-10.16)	<.001	135 (11.6)		73 (24.3)		7.95 (5.06-12.47)	<.001
	Three	150 (6.3)	162 (29.2)	20.35 (14.00-29.57)	<.001	72 (6.2)		77 (25.6)		15.30 (9.29-25.22)	<.001
	All	66 (2.8)	123 (22.2)	35.28 (22.44-55.47)	<.001	22 (1.9)		58 (19.3)		39.65 (20.69-76.00)	<.001

^a^aOR: adjusted odds ratio.

^b^After controlling for sex, age, education, smoking habit, and number of comorbidities.

^c^Ordinal variable summing up the presence of fever, myalgia, cough, and olfactory and taste disorders.

^d^N/A: not applicable.

**Table 5:** Age-specific adjusted odds ratios of positive molecular tests in nonhospitalized respondents with known molecular test results (n=4392, subsample B).

Symptom		Age <50 years (n=2659, 60.5%)						Age ≥50 years (n=1733, 39.5%)					
		Negative test (n=2176, 81.8%), n (%)		Positive test (n=483, 19.2%), n (%)		aOR^a,b^ (95% CI)	*P* value	Negative (n=1360, 78.5%), n (%)		Positive (n=373, 21.5%), n (%)		aOR (95% CI)	*P* value
Fever		341 (15.7)		239 (49.5)		1.98 (1.47-2.65)	<.001	177 (13.0)		205 (55.0)		3.46 (2.50-4.78)	<.001
Myalgia		599 (27.5)		308 (63.8)		1.61 (1.20-2.18)	.002	362 (26.6)		219 (58.7)		1.35 (0.98-1.86)	.07
Olfactory and taste disorders		177 (8.1)		323 (66.9)		15.88 (12.10-20.84)	<.001	114 (8.4)		184 (49.3)		5.25 (3.75-7.34)	<.001
Cough		639 (29.4)		266 (55.1)		1.14 (0.85-1.53)	.37	345 (25.4)		200 (53.6)		1.39 (1.02-1.91)	.04
Shortness of breath		223 (10.2)		117 (24.2)		0.90 (0.62-1.31)	.58	112 (8.2)		65 (17.4)		0.79 (0.51-1.23)	.29
Chest pain		267 (12.3)		133 (27.5)		0.95 (0.67-1.35)	.76	119 (8.8)		73 (19.6)		0.90 (0.59-1.38)	.63
Heart palpitations		243 (11.2)		101 (20.9)		0.80 (0.55-1.17)	.25	111 (8.2)		64 (17.2)		1.12 (0.73-1.73)	.60
Gastrointestinal disturbances		543 (25.0)		231 (47.8)		1.33 (1.00-1.76)	.048	274 (20.1)		151 (40.5)		1.14 (0.83-1.57)	.42
Conjunctivitis		198 (9.1)		80 (16.6)		0.93 (0.64-1.36)	.71	153 (11.3)		76 (20.4)		1.34 (0.92-1.97)	.13
Sore throat/rhinorrhea		918 (42.2)		272 (56.3)		0.89 (0.68-1.17)	.41	414 (30.4)		143 (38.3)		0.85 (0.63-1.16)	.31
Headache		837 (38.5)		300 (62.1)		1.23 (0.92-1.63)	.16	376 (27.6)		185 (49.6)		1.21 (0.88-1.65)	.24
**Number of symptoms^c^**													
	None	1167 (53.6)		52 (10.8)		1	N/A^d^	764 (56.2)		66 (17.7)		1	N/A^d^
	One	521 (23.9)		80 (16.6)		3.70 (2.54-5.41)	<.001	333 (24.5)		53 (14.2)		1.83 (1.23-2.73)	.003
	Two	285 (13.1)		101 (20.9)		8.91 (6.03-13.15)	<.001	156 (11.5)		84 (22.5)		6.20 (4.14-9.30)	<.001
	Three	147 (6.8)		146 (30.2)		24.39 (16.19-36.75)	<.001	75 (5.5)		93 (24.9)		13.98 (8.92-21.90)	<.001
	All	56 (2.6)		104 (21.5)		45.86 (27.94-75.29)	<.001	32 (2.4)		77 (20.6)		26.27 (14.95-46.17)	<.001

^a^aOR: adjusted odds ratio.

^b^After controlling for sex, age, education, smoking habit, and number of comorbidities.

^c^Ordinal variable summing up the presence of fever, myalgia, cough, and olfactory and taste disorders.

^d^N/A: not applicable.

After dichotomizing for the presence of two or more and of three or more symptoms, the resulting aORs were 12.17 (95% CI 9.50-15.59) and 22.44 (95% CI 16.93-29.75). When the four symptoms were singularly analyzed, a larger AUC (0.749, 95% CI 0.730-0.767) was found for olfactory and taste disorders, which were also characterized by a better specificity of 91.8%; however, myalgia showed higher sensitivity (61.6%) in classifying positive NPS tests. The combination of the four symptoms increased the AUC to 0.810 (95% CI 0.795-0.825), with higher sensitivity at the cutoff of two or more symptoms (70.7%) and higher specificity at the cutoff of three or more symptoms (91.2%) (data not shown).

As a final step, we quantified the number of probable SARS-CoV-2 infections in the nonhospitalized and nontested populations (subsample C) by calculating the frequencies for the combination of the four symptoms resulting from the analysis of subsample B. We found that 20,103 of the 165,782 respondents in subsample C (12.1%, 95% CI 12.0%-12.3%) had two or more symptoms suggestive of novel coronavirus disease and 7739 respondents (4.4%, 95% CI 4.3%-4.6%) had three or more symptoms, with accuracies of 77.2% and 83.0%, respectively.

## Discussion

### Principal Findings

This study, based on the responses of >170,000 persons to a web-based survey, outlined the COVID-19 symptom profiles of cases that did not require hospitalization during the outbreak of the epidemic in Italy. Olfactory and taste disorders, myalgia, fever, and cough are symptoms associated with laboratory-proven SARS-CoV-2 infection. Among 165,782 nonhospitalized and nontested respondents, 7739 to 20,103 (4.4% to 12.1%) experienced symptoms suggestive of COVID-19.

Although 102,973 of the 171,310 respondents (60.1%) reported at least one symptom compatible with viral infection, only 3.4% of these respondents had access to NPS testing for SARS-CoV-2. Respondents with at least one symptom accounted for 1065/1135 (93.8%) of patients with positive NPS tests, 2550/3650 (69.9%) of patients with negative NPS tests, and 396/532 (74.4%) of patients with unknown NPS test results. We here report that subgroups with symptomatology similar to that of people with positive NPS tests were not tested; this is a worrying finding that suggests that a large number of cases remained undiagnosed or were not correctly quarantined [19]. Active case finding with prompt isolation and contact tracing is a highly important means of ending the spread of SARS-CoV-2 infection [20], which otherwise is likely to continue through households [21]. The very limited number of respondents who were diagnosed based on NPS testing is a consequence of the decision by health authorities to reserve the use of diagnostics for clinically severe cases, thus creating suboptimal conditions for effective contact tracing.

A number of papers have described the clinical characteristics, symptoms, and disease course of inpatients [22,23] and outpatients [24] with SARS-CoV-2; however, little is still known about the natural history of the infection and its clinical spectrum or rate of symptoms in nonhospitalized cases with COVID-19. In our analyses, we showed a strong association between olfactory and taste disorders and positive NPS tests; respondents with positive NPS tests had a more than 10-fold increased risk of having olfactory and taste disorders. In line with our findings, olfactory and taste disorders have been reported to be symptoms specific of SARS-CoV-2 infection in clinical [8,25] and nonclinical [9,26,27] settings. Among 18,401 users of a COVID symptom tracker mobile app in the United Kingdom and United States who underwent molecular testing, loss of smell in addition to fever and persistent cough were found to be potential predictors of COVID-19 [9]. Similar results were recently reported from two other web-based surveys of Italian [26] and French [27] populations. Consistent with the aforementioned population studies, we also found that other COVID-19–related symptoms as fever, myalgia, or cough were significantly associated with positive NPS test results, although the association was less specific than that of olfactory and taste disorders. Overall, the four above-mentioned symptoms demonstrated an additive effect that increases the probability of a positive NPS test.

Interestingly, our subset analyses revealed some associations between the respondents’ symptoms and their demographic characteristics. The association between olfactory and taste disorders and positive NPS test results was stronger in younger patients, possibly because the known deterioration in the sense of smell during aging [28] means that younger respondents are more likely to notice its loss. We also found that positive NPS test results were more closely associated with olfactory and taste disorders in women and with fever in men, although both symptoms were significantly associated with positive NPS tests in both sexes. An association between female sex and olfactory and taste disorders has also been reported in hospitalized COVID-19 patients [8].

Notably, in the subpopulation of 165,782 participants who had not undergone NPS testing and were nonhospitalized, we calculated with an accuracy close to 80% that 12.1% of these participants had two or more of these symptoms and 4.4% had three or more, indicating a substantial number of adults with COVID-19–like illness. Applying the most conservative criterion (presence of three or more symptoms at the same time), characterized by a specificity of 91.2%, we estimated that about 2.2 million Italian adults had high probability of being symptomatic for COVID-19 up to April 21, 2020.

The estimation of the real proportion of the infected population is a fundamental indicator for public health policy makers in the ongoing COVID-19 pandemic. During the epidemic peak, model-based estimates [29] suggested that the ratio of notified to actual cases ranged from 1:5 to 1:20. However, to date in Italy, real-world data have been limited to restricted local settings or have only been available in the case of NPS testing of symptomatic patients with serious illness who require intensive or subintensive medical care. This lack has led to a wide underestimation of the spread of COVID-19 in mildly symptomatic individuals or in those with limited access to testing. Our results appear to be quite consistent with those of other surveys performed in large populations. A model that combined symptoms to predict probable infection was applied to the data derived from the COVID symptom tracker mobile app in the United Kingdom and United States [9], and the results indicated that 17.4% of users were likely to have COVID-19–like infection. Data from a nationally representative survey in Canada indicated that approximately 8% of adults reported that they or someone in their household had symptoms suggestive of COVID-19 in March 2020 [10].

These findings suggest that during a pandemic, when testing and contact tracing should be prioritized, the presence of such symptoms, also detected through a simple anamnestic investigation, may be an early indicator of SARS-CoV-2 infection in individuals who should be quarantined and molecularly tested.

It is also interesting to note that 66/856 (7.7%) of nonhospitalized patients with a positive NPS test reported no symptoms. A number of studies have suggested that asymptomatic patients can spread the virus [30,31]. According to the results of 16 SARS-CoV-2 testing studies pooled by Oran and colleagues, asymptomatic persons accounted for approximately 40% to 45% of COVID-19 infections [6]. In an Italian population study carried out on about 2500 residents in the municipality of Vò, the authors showed that the age-adjusted prevalence of COVID-19 asymptomatic cases was 43.2% (95% CI 32.2%-54.7%) [5]. Due to the characteristics of our study, it is unsuitable for precisely estimating the percentage of completely asymptomatic individuals, and our lower-than-expected findings can be explained by the limited access to molecular testing for asymptomatic individuals and by the possible overreporting of symptoms.

Our data concerning an apparently protective role of smoking in relation to positive NPS test results add new evidence to a panorama in which it has been suggested that this habit may have divergent clinical, prognostic, and epidemiological effects in patients with COVID-19 [32]. This issue will be investigated in more detail in a separate paper to contribute further to the current debate [33].

### Study Limitations and Strengths

Given the voluntary nature of the survey, it was not intended to assess a representative sample of the general population. However, extensive participation allowed us to collect a sample that is quite balanced although it is more shifted toward women and younger respondents with a higher level of education, as can be expected from a web-based questionnaire. The characteristics of a web-based survey may have also introduced a bias that led people with symptoms to respond more often than those without symptoms, and people who are health-conscious may have exaggerated (overreported) their symptoms. In addition, some symptoms (eg, olfactory and taste disorders) are more likely to be subject to recall bias due to media emphasis on their association with the disease.

At the date of the survey collection, the NPS testing rate among Italian adults (age ≥18 years) was estimated to be 1.92% [3], versus 3.10% among responders to the EPICOVID19 survey; this is suggestive of a greater propensity to participate for individuals who felt at higher risk, for symptomatology or closeness to COVID-19 cases. On April 21, the total number of SARS-CoV-2 cases in Italy was 183,957 [3] of the 971,246 individuals who underwent the NPS test, with an NPS-positive cumulative prevalence rate of 18.9%, similar to the rate of 23.7% observed in our study. By that time, the cumulative number of hospitalized patients with COVID-19 in Italy was 78,205, and the number of deceased due to COVID-19 (unknown if hospitalized) was 24,648. The cumulative prevalence rate of hospitalized COVID-19 cases therefore ranged from 0.15% to 0.20% among Italian adults. The total number of EPICOVID19 respondents who were hospitalized for suspected or confirmed COVID-19 illness was 610/171,310 (0.36%); among these patients, 279 (0.16%) had positive NPS tests, in line with the hospitalization rates in the general population.

As the sample was self-selected, our results should be generalized with caution. Finally, a single self-reported negative test cannot exclude a possible SARS-CoV-2 infection.

Web-based surveys have become an accepted, low-cost, and scalable means of efficiently and rapidly involving a large number of people in a study regardless of geographical distance [34,35]; therefore, they are preferable to more traditional, time-consuming, and expensive methods, especially in an ongoing emergency situation. Further, in the context of this outbreak, the EPICOVID19 survey may have included people who have had no other opportunity to report their symptoms. It is noteworthy that our survey achieved satisfactory geographical coverage; as expected, the coverage was proportional to the distribution of COVID-19 infection and to the reasonable likelihood that communities living in more affected areas would be more willing to respond.

To the best of our knowledge, this is the largest Italian web-based survey of SARS-CoV-2 symptoms; notably, it was carried out during the peak of the epidemic in Italy, when data at the population level were unavailable. National authorities, health care workers, and the public have received little information about the real spread of the infection since it started. Our preliminary findings shed some light on paucisymptomatic or mild infections with COVID-19 in Italy.

### Conclusions

The adoption of effective strategies and ready-to-use digital tools such as the real-time reporting internet-based survey EPICOVID19 to ascertain the positivity of paucisymptomatic carriers is still urgently needed in Italy and worldwide. The implementation of these strategies is also fundamental in countries, like those in Europe, where the spread of the infection is currently declining but where programs of active surveillance are necessary to reduce the risk of a new SARS-CoV-2 outbreak in the future. Many individuals with COVID-like infection are destined to remain beyond the control of health authorities, thus representing an important source of further spread of the infection. The determination of a symptomatic profile capable of easily identifying a suspected case may greatly contribute to containing the pandemic. Although they are also associated with other respiratory tract infections, the simultaneous presence of symptoms such as fever, cough, myalgia, and olfactory and taste disorders revealed by this study appears to be associated with a high probability of carrying active SARS-CoV-2 infection in a pandemic context.

## Appendix

Multimedia Appendix 1 EPICOVID19 web-based survey questionnaire.

Multimedia Appendix 2 Supplementary tables.

## Abbreviations

aOR: adjusted odds ratio
AUC: area under the curve
COVID-19: coronavirus disease
EU GDPR: European Union General Data Protection Regulation
MERS: Middle Eastern respiratory syndrome
NPS: nasopharyngeal swab
ROC: receiver operating characteristic
SARS: severe acute respiratory syndrome
SARS-CoV-2: severe acute respiratory syndrome coronavirus 2
