# Supplementary material for: Self-Reported Symptoms of SARS-CoV-2 Infection in a Nonhospitalized Population in Italy: Cross-Sectional Study of the EPICOVID19 Web-Based Survey
Source: JMIR Public Health Surveill. 2020 Sep 18;6(3):e21866. doi: 10.2196/21866 (PMC7505691; doi:10.2196/21866)
Supplement: Multimedia Appendix 1 [file publichealth_v6i3e21866_app1.docx]

**SOCIO-DEMOGRAPHIC DATA**

**mandatory questions*

1. **Sex at birth*** M |__| F |__|
2. **Year of birth *** |__|__|__|__|
3. **Ethnicity***

|__| Europe

|__| Asia

|__| North America

|__| Central/South America

|__| Africa

|__| Oceania

|__| Other

1. **Postal code*** |__|__|__|__|__|
2. **Municipality***  |__|__|__|__|__|__|__|__|__|__|__|__|__|__|__|__|__|__|__|__|__|__|__|__|__|
3. **Education***

|__| None

|__| Primary school

|__| Middle high school

|__| High school

|__| University degree

|__| Post graduate degree (PhD, vocational master, medical specialization)

1. **Occupational status***

|__| Employed

|__| Student

|__| Unemployed

|__| Retired

|__| Other

1. **Last occupation***

|__| Armed forces occupations

|__| Managers

|__| Intellectual and scientific professionals (e.g. clinicians, engineers, researchers, teachers)

|__| [Technicians](https://en.wikipedia.org/wiki/Technician) and associate professionals (e.g. healthcare technicians, IT technicians)

|__| Clerk

|__| Service and sales workers (e.g. merchants, sales persons)

|__| Craft and related trades workers

|__| Plant and machine operators, assemblers, and drivers of vehicles

|__| Other

**CLINICAL EVALUATION**

1. **Did you have one or more of the following symptoms since the 1st of February 2020? ***

|__| Fever with a temperature greater than 37.5 °C for at least three consecutive days

|__| Cough

|__| Sore throat/rhinorrea

|__| Headache

|__| Myalgia

|__| Olfactory or taste disorders

|__| Shortness of breath

|__| Chest pain

|__| Feelings of having a fast-beating

|__| Gastrointestinal disorders (diarrhoea, nausea, vomiting)

|__| Conjunctivitis

|__| Pneumoniae

1. **If you had at least one of the symptoms above, please indicate in which month they occurred for the first time ***

|__| February

|__| March

|__| April

1. **Have you ever been diagnosed with one or more of the following conditions?**

|__| Lung diseases (e.g. asthma, obstructive pulmonary disease)

|__| Heart diseases (e.g. ischemic heart disease, atrial fibrillation)

|__| Hypertension

|__| Kidney diseases

|__| Immune system diseases (e.g. thyroid disease, psoriasis, rheumatoid arthritis)

|__| Tumours

|__| Metabolic diseases (e.g. diabetes, obesity, gout)

|__| Liver diseases (e.g. hepatitis, cirrhosis, liver failure)

|__| Depression and/or anxiety

1. **Please indicate other conditions**

|__| Surgical procedures under general anaesthesia during the last year

|__| Transplants

|__| Allergies

|__| Pregnancy

|__| Non self-sufficient in carrying out daily activities

|__| Healthcare workers (e.g. clinicians, nurses, rescuer, pharmacist)

1. **Did you carry out the following vaccinations?***

- Flu shot during the last autumn |__| Yes |__| No

- Anti-pneumococcal in the last 12 months |__| Yes |__| No

- Other vaccinations in the last 12 months |__| Yes |__| No

1. **Do you regularly take one or more of the following medicines?**

|__| Aspirin

|__| Anti-hypertensive

|__| Hypocholesterolemic drugs

|__| Anti-diabetics

|__| Anti-cancer drugs

|__| Corticosteroids

|__| Thyroid drugs (e.g. euthyrox)

|__| Anti-inflammatory drugs

|__| Anxiety medications and/or sedatives

|__| Anti-depressant

|__| Supplements (e.g. vitamins)

*If females*

1. **Have you ever been taking birth pills and/or hormone replacement therapy?***

|__| No

|__| Yes, in the past, for less than 5 years

|__| Yes, in the past, for more than 5 years

|__| Yes currently, taking it less than 5 years

|__| Yes currently, taking it more than 5 years

1. **Indicate the number of completed pregnancies***

|__| 0

|__| 1

|__| 2

|__| 3 or more

1. **Have you been in a close contact (direct contact at a distance of less than 2 meters, or in a closed environment such as a house, workplace, transportation vehicles) with confirmed COVID-19 cases, live or deceased?***

|__| Yes |__| No

1. **Have you been in a close contact (direct contact at a distance of less than 2 meters, or in a closed environment such as a house, workplace, transportation vehicles) with suspected COVID-19 cases, live or deceased?***

|__| Yes |__| No |__| I don’t know

1. **Did you contact the emergency number and/or the general practitioner to report any symptoms of suspected infection by COVID-19?***

|__| No

|__| No but I went to the hospital on my own initiative

|__| Yes, and they suggested to me isolation

|__| Yes, and they did not suggest to me isolation

|__| Yes, and I was sent to the hospital

1. **Have you been tested for COVID-19? ***

|__| Yes, with a positive result

|__| Yes, with a negative result

|__| Yes, but I do not know the result

|__| No, I did not perform any test

1. **Have you been hospitalized due to COVID-19, either as a suspected or as a confirmed case?***

|__| Yes |__| No

1. **Please indicate other elements that might be of relevance for COVID-19** __________________________________________________________________________________________________________________________________________________________________________

**PERSONAL CHARACTERISTICS AND HEALTH STATUS**

1. **How would you describe your health in general?***

|__| Very bad |__| Bad |__| Adequate |__| Good |__| Very good

1. **Do you fear getting infected with the coronavirus (COVID-19)?***

|__| No

|__| Just a little bit

|__| Neutral

|__| Quite enough

|__| Yes, a lot

1. **Do you fear your family being infected with the coronavirus (COVID-19)?***

|__| No

|__| Just a little bit

|__| Neutral

|__| Quite enough

|__| Yes, a lot

**HOUSING CONDITIONS**

1. **Your home is located in**:*

|__| City centre with more than 100.000 inhabitants

|__| Suburbs of cities with more than 100.000 inhabitants

|__| Small town

|__| Countryside

1. **Your home is located in an area where the road traffic is**:*

|__| Intense (living near a busy road)

|__| Moderate

|__| Low

1. **How many rooms there are in your home (excluding the bathroom and auxiliary spaces)?***

|__| One |__| Two |__| Three |__| More than three

1. **Besides you, how many people live in your household?***

|__| None |__| One |__| Two |__| More than two

1. **Are there in the same household elderly persons or anyone with immunocompromising or chronic disease conditions?***

|__| Yes |__| No

**LIFESTYLE**

1. **How many people have you been in contact with in average, prior to the Governmental restrictions on lockdown?***

|__| Less than 10 |__| Between 10 and 100 |__| More than 100

1. **Do you smoke?***

|__| I have never smoked or I smoked less than 100 cigarettes in my lifetime

|__| I am a former smoker (I have smoked at least 100 cigarettes in my lifetime and I do not smoke anymore)

|__| Yes, smoking less than 10 cigarettes per day

|__| Yes, smoking between 10 and 20 cigarettes per day

|__| Yes, smoking more than 20 cigarettes per day (more than one pack per day)

*If former smoker*

1. **How many year have you smoked?*** |__|__|

*If current smoker*

1. **How many years?*** |__|__|
2. **Prior to the lockdown by the Governmental restrictions, for how long did you follow a regular routine of moderate or intense physical activity (swimming, racing)?***

|__| Not doing any physical activity or doing it for less than 10 minutes per week

|__| Between 10 minutes and two hours and half per week

|__| More than two hours and half per week

**BEHAVIOURS FOLLOWING THE LOCKDOWN**

1. **Please indicate your employment status following the restrictions introduced by the Italian Government since March, 9th 2020 ***

|__| Continued going to work at my workplace

|__| Working from home

|__| I had to stop working due to the emergency

|__| I am not employed

1. **Since March, 9th 2020, how many times do you go out during a week? ***

|__| Never

|__| 1

|__| 2-3

|__| 4-5

|__| 6 or more

1. **Since March, 9th 2020, do you use public transport go to work or to provide supplies***

|__| No, never

|__| Yes, 1-3 times per week

|__| Yes, 4-6 times per week

|__| Yes, 7 or more times per week

**Any additional information and comments**

__________________________________________________________________________________________________________________________________________________________________________________________________________________________________________
