# Supplementary material for: Self-Reported Symptoms of SARS-CoV-2 Infection in a Nonhospitalized Population in Italy: Cross-Sectional Study of the EPICOVID19 Web-Based Survey
Source: JMIR Public Health Surveill. 2020 Sep 18;6(3):e21866. doi: 10.2196/21866 (PMC7505691; doi:10.2196/21866)
Supplement: Multimedia Appendix 2 [file publichealth_v6i3e21866_app2.docx]

**Supplementary Table S1. Characteristics of the respondents by molecular test for SARS-CoV-2 (N=171,310, sample A)**

|  | **Not done** | | **Performed, with a negative result** | | **Performed, with a positive result** | | **Performed, with an unknown result** | |  |
| --- | --- | --- | --- | --- | --- | --- | --- | --- | --- |
|  | **n** | **%** | **n** | **%** | **n** | **%** | **n** | **%** | ***P*-value** |
| Sex at birth |  |  |  |  |  |  |  |  |  |
| Females | 99,084 | 59.7 | 2,440 | 66.8 | 668 | 58.9 | 351 | 66.0 | <.001 |
| Males | 66,909 | 40.3 | 1,210 | 33.2 | 467 | 41.1 | 181 | 34.0 |  |
| Age class |  |  |  |  |  |  |  |  |  |
| 18-30 | 21,506 | 13.0 | 465 | 12.7 | 108 | 9.5 | 70 | 13.2 | <.001 |
| 30-39 | 33,163 | 20.0 | 835 | 22.9 | 222 | 19.6 | 133 | 25.0 |  |
| 40-49 | 36,053 | 21.7 | 932 | 25.5 | 226 | 19.9 | 108 | 20.3 |  |
| 50-59 | 37,387 | 22.5 | 915 | 25.1 | 317 | 27.9 | 137 | 25.8 |  |
| 60-69 | 27,095 | 16.3 | 437 | 12.0 | 193 | 17.0 | 73 | 13.7 |  |
| 70-79 | 9,206 | 5.5 | 56 | 1.5 | 53 | 4.7 | 9 | 1.7 |  |
| 80+ | 1,583 | 1.0 | 10 | 0.3 | 16 | 1.4 | 2 | 0.4 |  |
| Education |  |  |  |  |  |  |  |  |  |
| Primary school or less | 8,861 | 5.3 | 110 | 3.0 | 49 | 4.3 | 21 | 3.9 | <.001 |
| Middle or high school | 56,576 | 34.1 | 657 | 18.0 | 319 | 28.1 | 134 | 25.2 |  |
| University degree or post-graduate | 100,556 | 60.6 | 2,883 | 79.0 | 767 | 67.6 | 377 | 70.9 | <.001 |
| Occupational status |  |  |  |  |  |  |  |  |  |
| Unemployed | 7,675 | 4.6 | 50 | 1.4 | 26 | 2.3 | 17 | 3.2 |  |
| Employed | 114,918 | 69.2 | 3,296 | 90.3 | 921 | 81.1 | 450 | 84.6 |  |
| Retired | 22,616 | 13.6 | 112 | 3.1 | 120 | 10.6 | 27 | 5.1 |  |
| Student | 11,795 | 7.1 | 108 | 3.0 | 24 | 2.1 | 26 | 4.9 |  |
| Other | 8,989 | 5.4 | 84 | 2.3 | 44 | 3.9 | 12 | 2.3 |  |
| Smoking habit |  |  |  |  |  |  |  |  |  |
| Never smokers | 95,068 | 57.3 | 2,250 | 61.6 | 743 | 65.5 | 320 | 60.2 |  |
| Former smokers | 39,843 | 24.0 | 757 | 20.7 | 284 | 25.0 | 119 | 22.4 |  |
| Current smokers | 31,082 | 18.7 | 643 | 17.6 | 108 | 9.5 | 93 | 17.5 | <.001 |
| Number of co-morbidities |  |  |  |  |  |  |  |  |  |
| None | 107,744 | 64.9 | 2,389 | 65.5 | 705 | 62.1 | 343 | 64.5 |  |
| One | 43,205 | 26.0 | 926 | 25.4 | 317 | 27.9 | 130 | 24.4 |  |
| Two | 11,543 | 7.0 | 265 | 7.3 | 86 | 7.6 | 46 | 8.6 |  |
| Three or more | 3,501 | 2.1 | 70 | 1.9 | 27 | 2.4 | 13 | 2.4 | .46 |
| Hospitalized for suspected/confirmed SARS-CoV-2 infection | 211 | 0.1 | 114 | 3.1 | 279 | 24.6 | 6 | 1.1 | <.001 |
| **All** | **165,993** | **96.9^a^** | **3,650** | **2.1^a^** | **1,135** | **0.7^a^** | **532** | **0.3^a^** |  |

*^a^Row percentage*

*If unspecified, percentages are column%.*

**Supplementary Table S2. Tetrachoric correlation matrix of symptoms in non-hospitalized respondents with known molecular test results (n=4,392, subsample B)**

|  | **Fever** | **Headache** | **Myalgia** | **Olfactory and taste disorders** | **Shortness of breath** | **Chest pain** | **Heart palpitations** | **Gastro-intestinal disturbances** | **Conjunctivitis** | **Sore throat/ rhinorrhea** |
| --- | --- | --- | --- | --- | --- | --- | --- | --- | --- | --- |
| **Fever** | 1 |  |  |  |  |  |  |  |  |  |
| **Headache** | 0.412 | 1 |  |  |  |  |  |  |  |  |
| **Myalgia** | 0.647 | 0.587 | 1 |  |  |  |  |  |  |  |
| **Olfactory and taste disorders** | 0.613 | 0.430 | 0.601 | 1 |  |  |  |  |  |  |
| **Shortness of breath** | 0.463 | 0.388 | 0.486 | 0.429 | 1 |  |  |  |  |  |
| **Chest pain** | 0.445 | 0.445 | 0.545 | 0.434 | 0.618 | 1 |  |  |  |  |
| **Heart palpitations** | 0.378 | 0.419 | 0.442 | 0.346 | 0.578 | 0.580 | 1 |  |  |  |
| **Gastrointestinal disturbances** | 0.419 | 0.468 | 0.499 | 0.451 | 0.421 | 0.430 | 0.429 | 1 |  |  |
| **Conjunctivitis** | 0.219 | 0.356 | 0.379 | 0.290 | 0.302 | 0.303 | 0.293 | 0.318 | 1 |  |
| **Sore throat/rhinorrhea** | 0.236 | 0.420 | 0.378 | 0.298 | 0.327 | 0.332 | 0.286 | 0.345 | 0.304 | 1 |
| **Cough** | 0.602 | 0.409 | 0.510 | 0.471 | 0.562 | 0.492 | 0.390 | 0.361 | 0.285 | 0.488 |

*For all coefficients, P-values are <.001*

**Supplementary Table S3. Sensitivity analysis: adjusted odds ratios^a^ of positive molecular test in non-hospitalized respondents with known test results, excluding those who reported that the onset of signs/symptoms occurred in February 2020 (n=3,165)**

|  | **Negative** | | **Positive** | |  |  |
| --- | --- | --- | --- | --- | --- | --- |
|  | **n=2,418** | **pct=76.4^b^** | **n=747** | **pct=23.6^b^** | **aOR (95% CI)** | ***P*-value** |
| Fever | 295 | 12.2 | 391 | 52.3 | 2.75 (2.13-3.55) | <.001 |
| Myalgia | 252 | 10.4 | 175 | 23.4 | 1.42 (1.10-1.83) | .008 |
| Olfactory and taste disorders | 551 | 22.8 | 462 | 61.8 | 9.65 (7.61-12.24) | <.001 |
| Cough | 472 | 19.5 | 397 | 53.1 | 1.58 (1.23-2.03) | <.001 |
| Shortness of breath | 203 | 8.4 | 456 | 61.0 | 0.78 (0.57-1.08) | .14 |
| Chest pain | 213 | 8.8 | 156 | 20.9 | 0.83 (0.61-1.13) | .24 |
| Heart palpitations | 227 | 9.4 | 143 | 19.1 | 0.83 (0.60-1.14) | .25 |
| Gastrointestinal disturbances | 488 | 20.2 | 339 | 45.4 | 1.09 (0.86-1.40) | .48 |
| Conjunctivitis | 188 | 7.8 | 129 | 17.3 | 1.24 (0.90-1.70) | .18 |
| Sore throat/rhinorrhea | 649 | 26.8 | 355 | 47.5 | 1.08 (0.86-1.37) | .51 |
| Headache | 689 | 28.5 | 422 | 56.5 | 1.32 (1.03-1.68) | .03 |
| *Number of symptoms^c^* |  |  |  |  |  |  |
| None | 1,562 | 64.6 | 104 | 13.9 | 1 |  |
| One | 440 | 18.2 | 111 | 14.9 | 3.97 (2.92-5.38) | <.001 |
| Two | 230 | 9.5 | 161 | 21.6 | 11.03 (8.02-15.17) | <.001 |
| Three | 123 | 5.1 | 211 | 28.2 | 27.89 (19.64-39.59) | <.001 |
| All | 43 | 2.6 | 160 | 21.4 | 43.66 (28.69-66.44) | <.001 |

*^a^After controlling for sex, age, education, smoking habit, and number of co-morbidities*

*^b^Row percentage*

*^c^Ordinal variable summing up the presence of fever, myalgia, cough and olfactory and taste disorders*

*If unspecified, percentages are column%.*
